# Cigarette smoke challenges bone marrow mesenchymal stem cell capacities in guinea pig

**DOI:** 10.1186/s12931-017-0530-0

**Published:** 2017-03-23

**Authors:** Olga Tura-Ceide, Borja Lobo, Tanja Paul, Raquel Puig-Pey, Núria Coll-Bonfill, Jéssica García-Lucio, Valérie Smolders, Isabel Blanco, Joan A. Barberà, Víctor I. Peinado

**Affiliations:** 1 Department of Pulmonary Medicine, Hospital Clínic-Institut d’Investigacions Biomèdiques August Pi i Sunyer (IDIBAPS)-University of Barcelona, Villarroel, 170, Barcelona, 08036 Spain; 2 Biomedical Research Networking Center in Respiratory Diseases (CIBERES), Madrid, Spain

**Keywords:** Cigarette smoke, Chronic obstructive pulmonary disease, Mesenchymal stem cells, Cell homing-chemokines

## Abstract

**Background:**

Cigarette smoke (CS) is associated with lower numbers of circulating stem cells and might severely affect their mobilization, trafficking and homing. Our study was designed to demonstrate in an animal model of CS exposure whether CS affects the homing and functional capabilities of bone marrow-derived mesenchymal stem cells (BM-MSCs).

**Methods:**

Guinea pigs (GP), exposed or sham-exposed to CS, were administered via tracheal instillation or by vascular administration with 2.5 × 10^6^ BM-MSCs obtained from CS-exposed or sham-exposed animal donors. Twenty-four hours after cell administration, animals were sacrificed and cells were visualised into lung structures by optical microscopy. BM-MSCs from 8 healthy GP and from 8 GP exposed to CS for 1 month were isolated from the femur, cultured in vitro and assessed for their proliferation, migration, senescence, differentiation potential and chemokine gene expression profile.

**Results:**

CS-exposed animals showed greater BM-MSCs lung infiltration than sham-exposed animals regardless of route of administration. The majority of BM-MSCs localized in the alveolar septa. BM-MSCs obtained from CS-exposed animals showed lower ability to engraft and lower proliferation and migration. In vitro, BM-MSCs exposed to CS extract showed a significant reduction of proliferative, cellular differentiation and migratory potential and an increase in cellular senescence in a dose dependent manner.

**Conclusion:**

Short-term CS exposure induces BM-MSCs dysfunction. Such dysfunction was observed in vivo, affecting the cell homing and proliferation capabilities of BM-MSCs in lungs exposed to CS and in vitro altering the rate of proliferation, senescence, differentiation and migration capacity. Additionally, CS induced a reduction in CXCL9 gene expression in the BM from CS-exposed animals underpinning a potential mechanistic action of bone marrow dysfunction.

**Electronic supplementary material:**

The online version of this article (doi:10.1186/s12931-017-0530-0) contains supplementary material, which is available to authorized users.

## Background

Chronic obstructive pulmonary disease (COPD) is a multicomponent respiratory condition, often associated with significant extrapulmonary abnormalities, the so-called “systemic effects of COPD”. These extrapulmonary effects which include among others, systemic inflammation, weight loss and skeletal muscle dysfunction are clinically relevant. One additional extrapulmonary effect of COPD is bone marrow dysfunction [1]. Bone marrow is the human’s main reservoir of progenitor cells capable to be mobilized into the circulation in response to tissue damage. Progenitor cells present in the bone marrow are essential in tissue maintenance and restoration of the normal function by replacing terminally differentiated cells, lost as a consequence of physiological cell turnover or tissue damage [2]. COPD has been associated with reduced numbers of bone marrow derived circulating progenitor cells [3–8]. Disease severity, lower exercise capacity and airflow obstruction were also associated to a greater reduction in circulating progenitors [3, 5].

Within the bone marrow “niche” different subtypes of progenitor cells such as hematopoietic progenitor cells (HSCs) and bone marrow mesenchymal stem cells (BM-MSCs) synergize [9]. BM-MSCs protect HSCs integrity, providing an important microenviromental support and controlling the balance between HSC self-renewal, differentiation and proliferation. BM-MSCs have the ability to migrate to sites of ischemic, inflammatory or mechanical injury and can affect tissue microenvironment via the secretion of soluble factors [10]. The mechanisms by which BM-MSCs home to tissue damage are not fully understood, but it is believed that chemokine receptors and their ligands play a crucial role [11]. Importantly, once recruited to the site of tissue damage, BM-MSCs contribute to the regeneration of mesenchymal tissues by differentiating into a variety of cell types, including osteoblasts, chondrocytes, myocytes, adipocytes, and many other cells types [12]. Studies of BM-MSCs have provided evidence of safety and efficacy in animal and human lung explant models of acute and fibrotic lung injuries as well as in asthma, bronchopulmonary dysplasia, COPD, sepsis, and other lung diseases [13, 14]. There is no clear evidence of the potential role of these cells on the regeneration of lung tissue in these diseases. However, growing evidence suggests that paracrine and immunomodulatory effects released by the BM-MSCs are likely the responsible factors of the beneficial effects of MSC therapy [13, 14].

Cigarette smoke (CS) is the primary cause of COPD in developed countries [15]. The molecular processes that link CS and extrapulmonary effects of COPD are still uncertain, but persistent inflammation is assumed to trigger the onset of COPD. Interestingly, it has been shown that the amount of cigarettes smoked inversely correlates with the number of bone marrow derived progenitor cells, the number of which increase rapidly following smoking cessation [7, 16].

We hypothesize that in COPD, CS exposure compromises BM-MSCs functional capabilities severely affecting their proliferation, homing and repair mechanisms. Our study was designed to demonstrate in an experimental model of CS exposure whether CS produces BM-MSC dysfunction. This study was performed by investigating functional properties of BM-MSCs after in vivo lung administration and by studying in vitro their proliferation, migration, senescence, differentiation potential and chemokine gene expression profile. Bone marrow dysfunction caused by constant CS exposure and failure of dysfunctional bone marrow progenitor cells to cope with external injuries or cell apoptosis may be key to an inefficient lung tissue healing and to the development of irreversible COPD.

## Methods

### In vivo characterization of mesenchymal stem cells

BM-MSCs were isolated from both femurs of 6 naive or 6 CS-exposed Dunkin Hartley male guinea pigs (500 g) as previously described [17]. Briefly, bone marrow was flushed from femurs and cultured with DMEM-LG medium supplemented with 10% FBS, 1% Penicillin-Streptomycin, 1% Amphotericin B and 1% L-Glutamine (200nM, Sigma-Aldrich, St. Louis, MO). 6 h after cell plating, the medium was replaced to discard non-adherent cells. Remaining adherent cells were cultured until characteristic fusiform colonies were observed and expanded until 80% confluent. Cells were stained with the guinea-pig specific pan-leukocyte marker CD45 (MCA 1130, Serotec, Kidlington, UK) and analyzed by flow cytometry as previously described [4].

### In vivo administration of MSCs

**Fig. 1:**
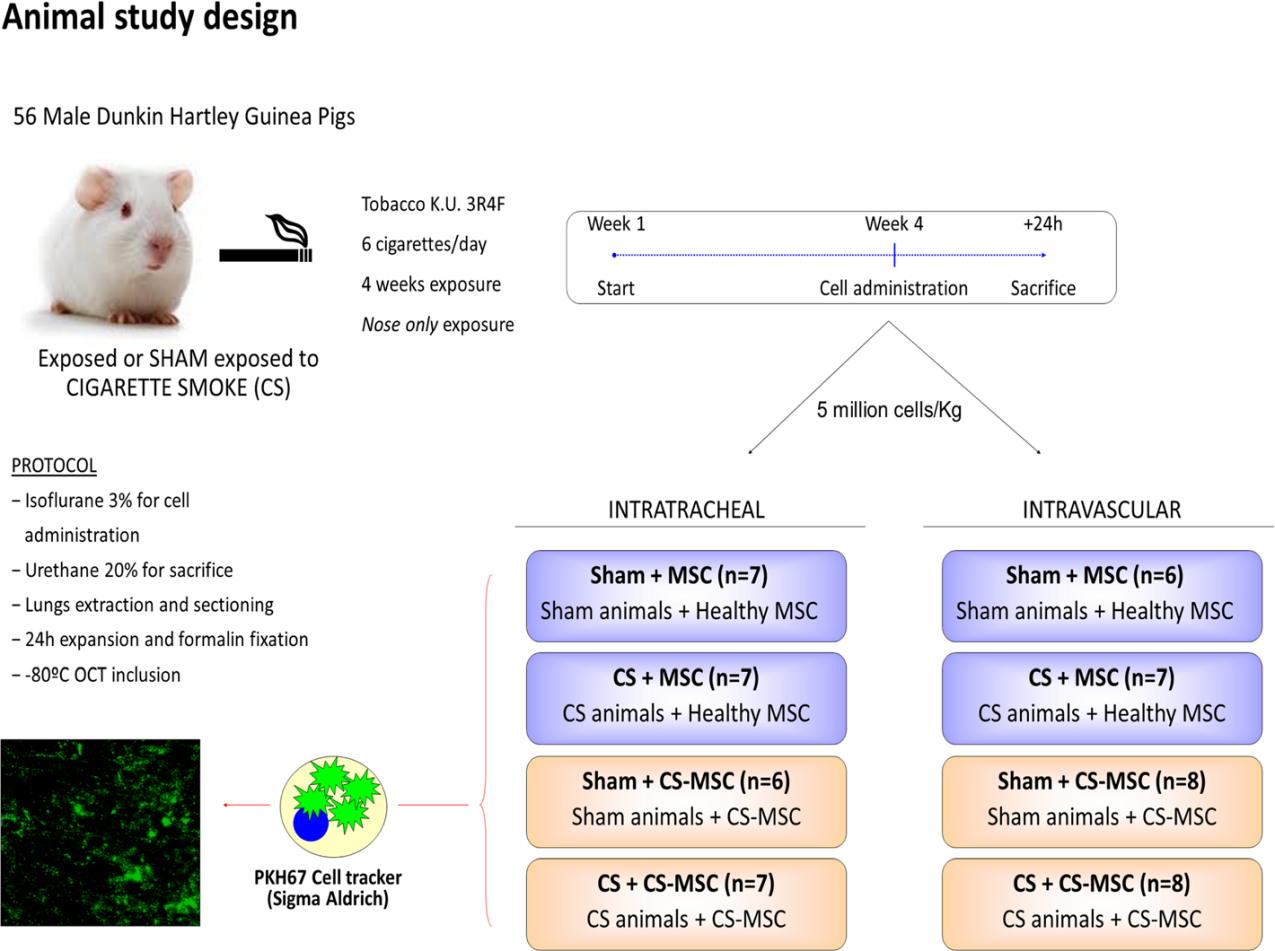
Schematic diagram of experimental design. Guinea pigs (starting ~ 500 g) were randomly distributed in 4 groups: Group 1; exposed and Group 2; sham-exposed to CS for subsequent BM-MSC intratracheal administration and Group 3; exposed and Group 4; sham-exposed to CS for BM-MSC vascular administration. Animals from CS groups were exposed to smoke generated by 6 non-filtered cigarettes for 4 weeks, 5 days/week using a nose-only system

Cell labeling was performed 24 h prior to administration. Cultured BM-MSCs were trypsinized, resuspended and incubated with the cytoplasmatic fluorescent cell tracker PKH67 (Sigma Aldrich) for 7 min in the dark. Fifty-seven Dunkin Hartley male guinea pigs were randomly distributed in 4 groups: Group 1; exposed to CS and Group 2; sham-exposed to CS for subsequent intratracheal administration and Group 3; exposed to CS and Group 4; sham-exposed to CS for subsequent vascular administration (Fig. 1). Animals from CS groups were exposed to smoke generated by 6 non-filtered cigarettes (3R4F, Kentucky University Research), 5 days/week during 4 weeks using a nose-only system (Protowerx Design Inc; Langley, British Columbia, Canada). 24 h after the last cigarette, all animals were anesthetized with 3% isofluorane for intratracheal instillation or with ketamine and xylacine for cardiac puncture administration of 5 × 10^6^ cells/kg BM-MSCs resuspended in 250 μl of PBS. 24 h after cell administration, all animals were sacrificed under urethane anesthesia and lung tissue was fixed in formalin under column pressure of 30 cm, included in OCT compound and stored at -80 °C. All procedures involving animal research were approved by the Ethics Committee of the University of Barcelona and were conducted following institutional guidelines that comply with international laws. In all cases, administration was well tolerated without mortality.

### In vitro characterization of mesenchymal stem cells

#### Adipogenic differentiation

Adherent cells derived from bone marrow were cultured in supplemented DMEM-LG medium until confluent. 3 days later, medium was replaced with Lonza Adipogenic differentiation medium, following the manufacturer’s instructions (rMSC Differentiation BulletKit®, Lonza). After 3 weeks, cultures were stained with Oil Red to verify the presence of lipidic vacuoles [18].

### Osteogenic differentiation

Adherent cells derived from bone marrow were seeded in fibronectin coated culture plates and cultured until 70% confluent in supplemented DMEM-LG medium. Thereafter, medium was replaced by supplemented DMEM-LG containing β-glycerophosphate (10 mM), L-ascorbic acid (50 μg/mL) and dexamethasone (10nM) to induce osteogenic differentiation. After 3 weeks, cultures were stained with Alizarin Red and Von Kossa stain for calcium deposits [18].

### Preparation of cigarette smoke extract (CSE)

Unfiltered mainstream smoke from 4 research grade cigarettes (3R4F, Kentucky University Research, Lexington, KY) was bubbled using a smoking apparatus into 50 ml of serum-free DMEM-LG and stored at -80 °C after sterile filtration through a 0.22 μm pore size filter.

### Cell growth kinetics and viability

Growth rate of BM-MSCs from sham or CS-exposed animals was assessed after 4, 8 and 12 days of culture. Expansion of cell subpopulations was calculated by dividing the final number of cells by the number of cells seeded at day 0 and expressed as percentage. For experiments with CSE cell growth was assessed after 4 days of culture and expressed as fold change. Cell viability was measured with a MTT-kit (Vybrant MTT Cell proliferation assay kit, Life Technologies) following the manufacturer’s instructions.

### Scratch wound healing assay

BM-MSCs from CS- or sham-exposed animals were cultured in 6-well plates with supplemented DMEM-LG medium. When confluent, a P200 tip was used to mark a vertical line down the middle of each well. During the next 24 h, pictures of each well were taken and the percentage of scratch occlusion was measured using ImagePro software (Media Cybernetics, Inc., Rockville, MD). For CSE-exposure in vitro experiments, BM-MSCs were cultured until confluent in a 24-well plate. As above, a P200 tip was used to make a straight scratch in the cell-layer of each well. Medium was immediately replaced by supplemented DMEM-LG medium with increasing concentrations of CSE (1/30, 1/20, 1/10 dilutions in growth medium) or vehicle and incubated for 30 h.

### Cellular senescence

10^3^ cells/well were seeded in a 48-well plate, and left to adhere overnight before medium was replaced and cells were incubated for 4 days with different concentrations of CSE (1/30, 1/20 and 1/10 dilutions) or vehicle. Then, cells were fixed and stained using Senescence Histochemical Staining Kit (Sigma-Aldrich) which measures the number of senescent cells based on a histochemical stain for β-galactosidase activity. Nuclei were counterstained with DAPI (Molecular Probes, Thermo Fisher Scientific, Waltham, MA). The percentage of senescent cells was assessed with ImageJ - Fiji software.

### RNA Isolation and Real Time PCR

For analysis of in vivo gene expression, lungs from sham and CS-exposed animals were extracted and stored in RNAlater (Ambion, Thermo Fisher Scientific, Waltham, MA). In a second set of experiments BM of guinea pigs exposed to CS for 6 months was obtained by flushing femurs and equally kept in RNAlater. For RNA isolation, tissue was homogenized (OmniTH international) and RNA was extracted using a column-based Clean-Up Kit following the manufacturer’s instructions (RNeasy Micro Kit, Qiagen, Hilden, Germany). For analysis of in vitro gene expression in response to CSE, 10 × 10^4^ cells per well were seeded in 6-well plates. After 24 h, culture medium was replaced with supplemented DMEM-LG medium with increasing concentrations of CSE or vehicle and cultured for additional 24 h until RNA extraction (TRIsure, Bioline GmbH, Luckenwalde, Germany). cDNA synthesis was performed with 1 μg of RNA with the High-Capacity cDNA Reverse Transcription Kit (Applied Biosystems, Foster City, CA, USA). Gene expression was measured by quantitative RT-PCR using DNA SYBR green I dye (SensiMixTM SYBR Hi-Rox, Bioline). The results were normalized to GAPDH or ß-Actin expression levels and relative gene expression was analyzed by the 2^-∆∆ct^-method [19].

### Statistical analysis

Results are expressed as mean ± SD or median and interquartile range, depending on the parametric or non-parametric distribution. Comparisons between groups were performed using an analysis of variance (ANOVA) or the Kruskal-Wallis test, when appropriate. Post hoc comparisons were done using Mann–Whitney *U*-test. Significant differences were considered at *p* < 0.05 level. All analyses were performed using SigmaPlot v.11.0 software (Systat Software Inc., Chicago, IL).

## Results

### Bone Marrow MSC isolation

**Fig. 2:**
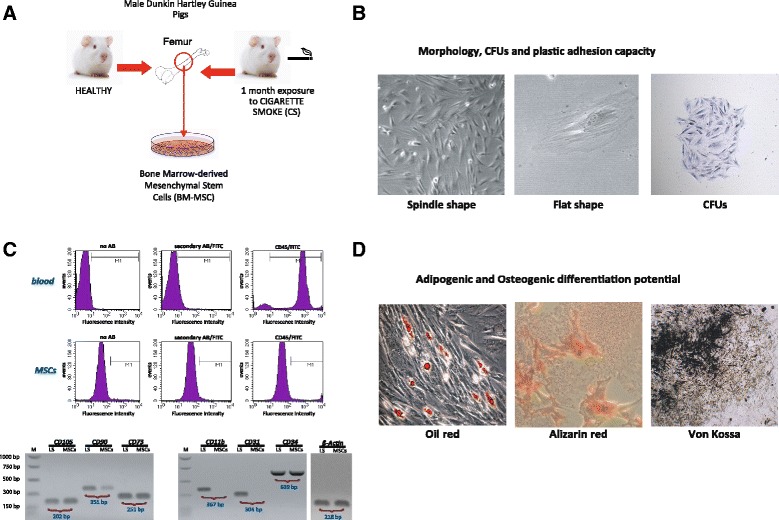
Validation of MSC properties. **a** BM-MSCs were harvested and cultured from guinea pigs’ bone marrow from healthy (sham-exposed) or CS-exposed individuals. **b** Cell characterization was determined by plastic adhesion, colony forming capacity, and morphology. **c** Purity was checked by flow cytometric analysis of CD45 expression in BM-MSCs and compared with whole blood. Fluorescence intensity of the no-antibody (*left panel*), the secondary antibody control (middle panel) and fluorescence intensity in the presence of the CD45 specific antibody (*right panel*) was measured. The lower panel shows gene expression analysis of positive (CD105, CD90, CD73) and negative (CD11b, CD31, CD34) MSC markers and ß-Actin. M: molecular weight; LS: lung tissue lysate. **d** Adipogenic and oesteogenic differentiation. Cells were incubated for 3 weeks in either induction or normal growth medium before von-Kossa and Alizarin red staining for calcium deposits or oil red to identify lipidic vacuoles

Cells obtained from guinea pigs’ bone marrow (Fig. 2a) showed typical BM-MSCs characteristics including plastic adhesion, colony forming capacity, spindle-shaped and flat morphology (Fig. 2b). BM-MSCs were CD45, CD11b and CD31 negative and positive for CD105, CD90, CD73 mesenchymal markers (Fig. 2c). When cultured in appropriate culture conditions BM-MSCs showed adipogenic and osteogenic potential (Fig. 2d).

### CS-exposed animals showed greater BM-MSC lung infiltration regardless of administration route

**Fig. 3:**
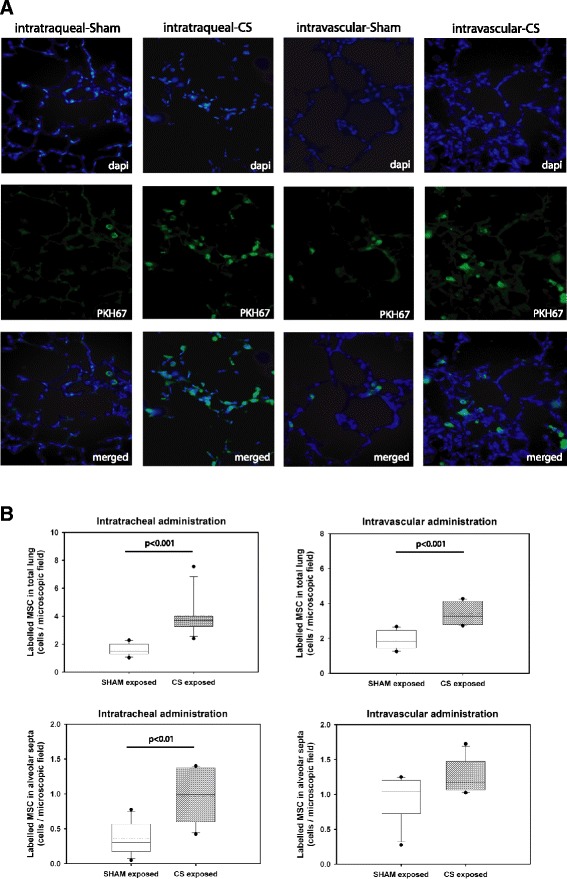
Identification and counting of tracked cells in lung sections under fluorescence microscope. **a** Lung tissue sections from sham or CS exposed animals 24 h after BS-MSC administration via intratracheal or intravascular route. Tissues were stained with DAPI (*blue*) for localization of nuclei. BM-MSCs were labelled with PKH67 (*green*) for cell tracking purposes. Identification and counting of tracked cells in the lung sections was assessed under a fluorescence microscope. Mean values from 20 photographed microscopic fields per animal were calculated. **b** Box plots of infiltrated BM-MSC in total lung and in the alveolar septa, *P* value was assessed by Mann–Whitney *U*-test

Lung sections from animals treated with exogenous administration of BM-MSCs were analyzed to determine their fate in vivo during the first 24 h post-administration. Regardless of administration route (intratracheal instillation or cardiac puncture), CS-exposed animals showed greater BM-MSC lung infiltration compared to sham-exposed animals (Fig. 3a-b) (*p* < 0.001) and Table 1. Moreover, we found that despite route of administration, these cells preferentially locate at the alveolar septum when compared to the number of cells found in the pulmonary arteries (*p* < 0.01) (Fig. 3b) and Table 1.

**Table 1:** Values of infiltrated labelled BM-MSCs in different lung structures administrated via intratracheal or intravascular route

	Experimental groups				*P* value Kruskall-Wallis
	ShA/HMSC	ShA/CSMSC	CSA/HMSC	CSA/CSMSC	
Intratracheal Administration					
MSC in total lung	1.450	1.688	3.700	3.05	<0.001
	1.294–1.996	1.525–1.725	3.25–3.975	2.569–3.256	
MSC in Alveolar septa	0.300	0.388	0.988	0.650	0.031
	0.175–0.569	0.375–0.500	0.600–1.375	0.350–0.825	
MSC in pulmonary arteries	0.000	0.037	0.000	0.00	0.641
	0.000–0.038	0.000–0.075	0.000–0.044	0.000–0.000	
Intravascular Administration					
MSC in total lung	1.813	1.337	3.264	2.612	<0.001
	1.450–2.450	1.125–1.663	2.792–4.105	2.362–3.003	
MSC in Alveolar Septa	1.038	0.200	1.175	0.463	<0.001
	0.725–1.200	1.125–0.225	1.063–1.475	0.362–0.700	
MSC in pulmonary arteries	0.025	0.075	0.125	0.087	0.362
	0.000–0.050	0.063–0.088	0.031–0.181	0.025–0.138	

### BM-MSCs isolated from CS-exposed animals have lower infiltration capability in vivo

**Fig. 4:**
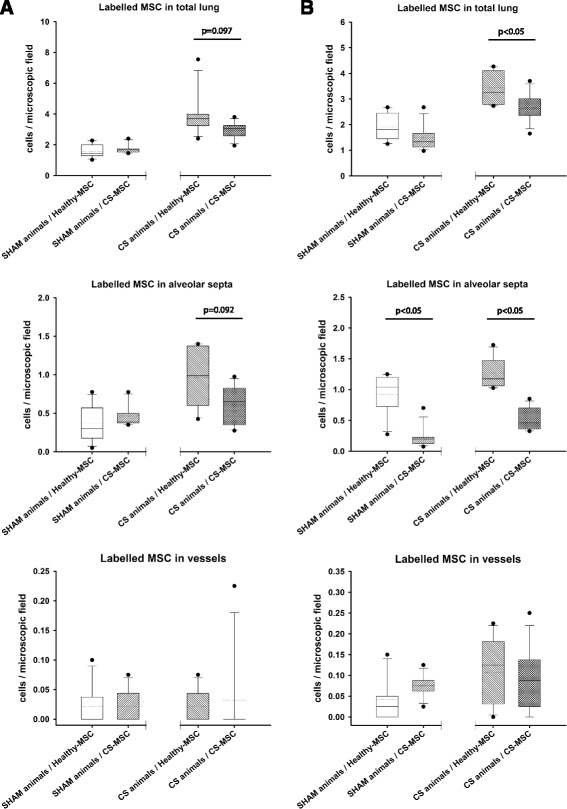
Box plots of infiltrated labelled cells in different lung structures. Cells were counted in total lung, alveolar septa and blood vessels after intratracheal (**a**) or vascular (**b**) administration of BM-MSC obtained from healthy or CS exposed animals, *P* values were obtained using the Mann–Whitney *U*-test

As above, CS-exposed animals showed greater CS-derived BM-MSC lung infiltration compared to lungs of sham-exposed animals regardless of administration route (Fig. 3) and Table 1. However, BM-MSCs isolated from CS-exposed animals had a reduced capacity to engraft into the recipient’s lung when compared to BM-MSCs isolated from sham-exposed GP. Yet again, these cells preferentially located at the alveolar septum, regardless the way of administration route (*p* < 0.05). Very few CS exposed or sham-exposed BM-MSCs administered by either route were localized in the adventitia of pulmonary vessels of sham or CS animals (Fig. 4a-b) and Table 1.

### CS exposure induces functional damage of BM-MSCs in vitro

**Fig. 5:**
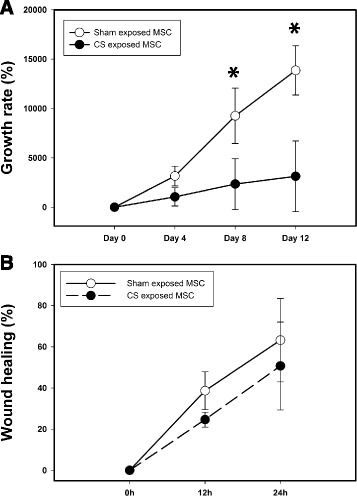
Cell growth kinetics of BM-MSCs. **a** Growth rate of BM-MSC obtained from healthy (*open dot*) or CS exposed (*black dot*) animals. Cell number was assessed after 4, 8 and 12 days of culture dividing the final number of cells by the number of cells plated at day 0. **b** Wound healing assay of BM-MSC obtained from healthy (*open dot*) or CS exposed (*black dot*) animals. Representative pictures were taken at 0, 12 and 24 h and the percentage of occlusion was calculated. All experiments were done in technical and biological triplicates. *P* value was assessed by Two way ANOVA Repeated Measures. *denotes *P* < 0.05

**Fig. 6:**
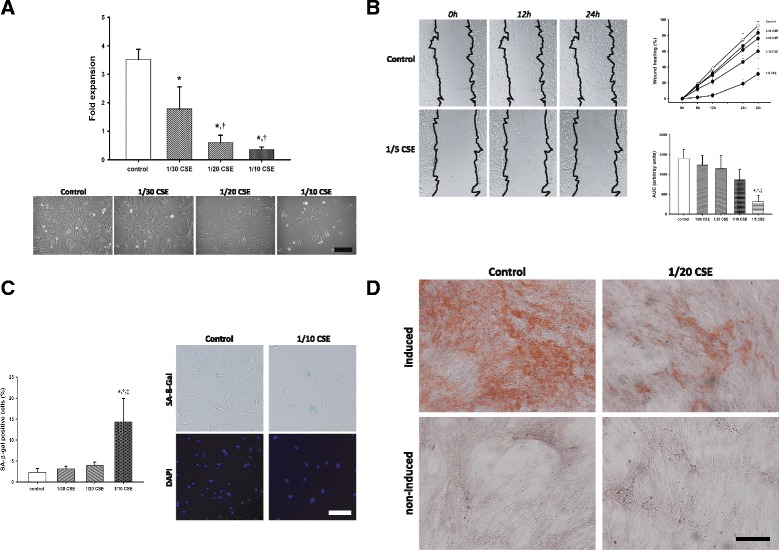
In vitro effects of CSE in cultured BM-MSC **a** BM-MSCs were incubated with increasing concentrations of CSE for 4 days. Bar graph shows the fold expansion of the cells in different conditions. In the lower panel are representative photomicrographs of the different conditions. Scale bar: 250 μm. **b** Wound healing assay: BM-MSCs were incubated with increasing concentrations of CSE after a scratch was performed in the confluent monolayer. Pictures were taken after 0, 6, 12, 24 and 30 h. The line graph shows wound closure kinetics and the bar graph shows the area under the curve (AUC) of the time curves under different conditions. **c** Cellular senescence assay in BM-MSCs after 24 h of stimulation with different concentrations of CSE. The bar graph shows the relative gene expression of SA-β-Gal expression. Scale bar: 150 μm. **d** Differentiation of BM-MSCs in presence or absence of CSE: Cells were incubated for 3 weeks in either induction or normal growth medium in the presence or absence of 5% CSE. Alizarin red stain. Scale bar 250 μm. *P* value was assessed by one-way ANOVA and post-hoc Holm-Sidak method. * *p* < 0.05 *vs* control; † *p* < 0.05 *vs* 1/30; ‡ *p* < 0.05 *vs* 1/20

To assess whether CS exposure had an effect on the functional characteristics of BM-MSCs, we determined both the proliferative, viability and migration capacities in vitro of BM-MSCs isolated from sham or CS-exposed animals. Results indicated that BM-MSCs from sham animals had a greater proliferation rate as compared to BM-MSCs from CS-exposed animals (*p* < 0.05) (Fig. 5a) and though did not reach statistical significance, sham-BM-MSCs showed higher migration capacity within the first 24 h, compared to CS exposed BM-MSC (Fig. 5b). BM-MSCs isolated from sham animals were exposed in vitro to increasing concentrations of CSE (1/30, 1/20, 1/10). Under these conditions, we determined their proliferation, viability, migration, senescence, apoptosis and osteogenic differentiation. Exogenous administration of CSE significantly reduced BM-MSCs proliferation compared to non-treated cells in a concentration dependent manner (Fig. 6a) (*p* < 0.001). This was most apparent for high concentrations of CSE (1/10). Cell viability was assessed after 24 h using MTT assay but no significant differences were observed between the groups (Additional file 1: Figure S1).

CSE exposure significantly reduced BM-MSCs migration potential in a dose-dependent manner (Fig. 6b) (*p* < 0.001). Quantification of the percentage of covered area over time showed that CSE-treated BM-MSCs had reduced closure kinetics during recovery from a scratch compared to non-treated cells (*p* < 0.001). The percentage of cells positive for SA-β-gal activity rose significantly at the highest concentration of CSE tested (1/10) (Fig. 6c). Caspase-3 levels were also measured in BM-MSCs treated with increasing concentrations of CSE (1/30, 1/20, 1/10). The differences observed in caspase-3 gene expression did not reach statistical significance (Additional file 1: Figure S1). Osteogenic differentiation was visibly diminished in BM-MSCs treated with CSE (Fig. 6d).

### BM tissue from CS-exposed animals showed a reduction of CXCL9 expression compared to sham-exposed animals

**Fig. 7:**
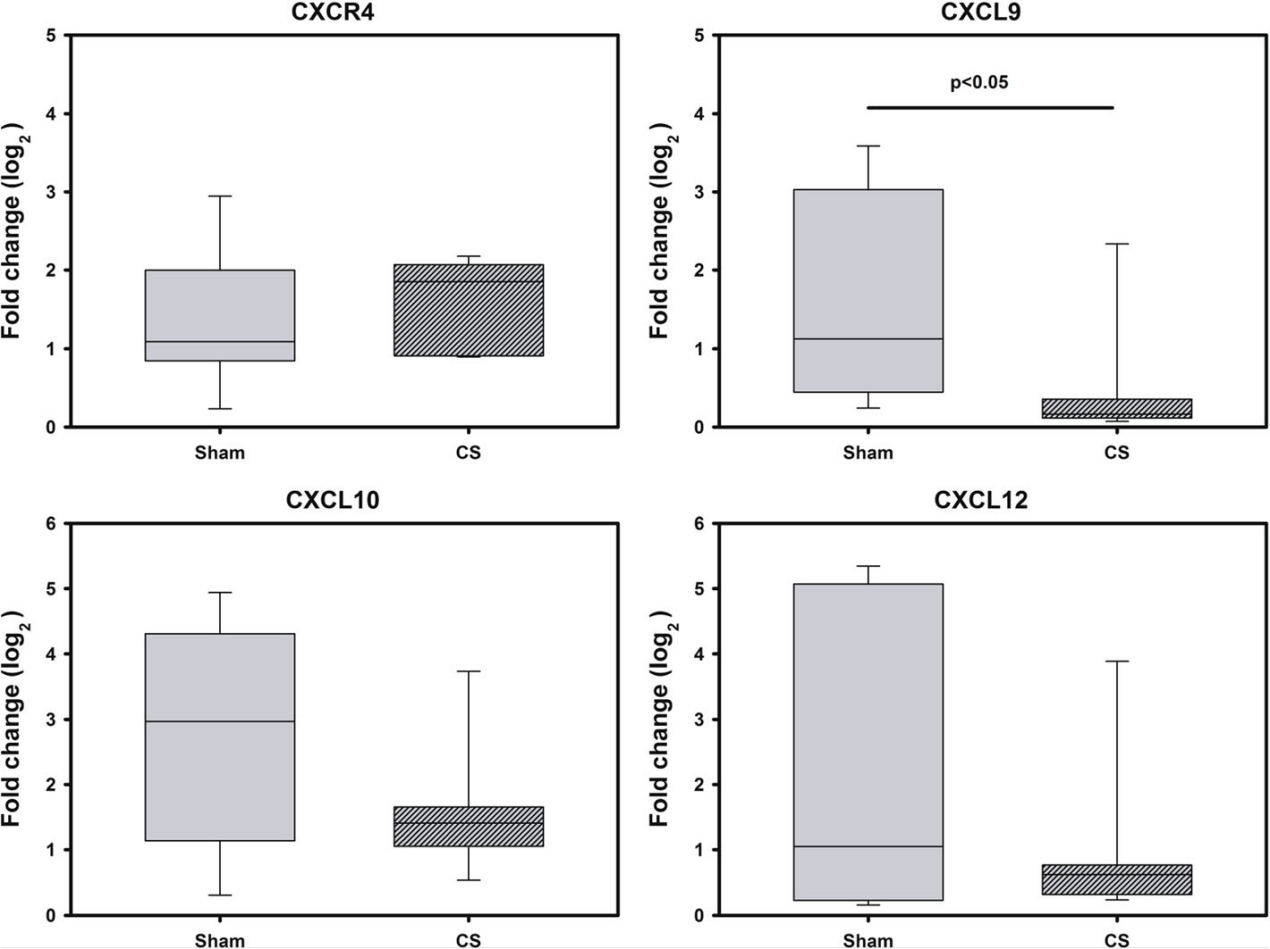
Gene expression of chemokines in BM homogenates of CS-exposed guinea pigs. The box plots show the median and the interquartile range of the relative gene expression of 6 animals. Reactions were run in triplicates Relative gene expression was analyzed by 2^-ΔΔct^-method. Significant differences were assessed using the Mann–Whitney *U*-test * *p* < 0.05 vs control

To evaluate the effect of CS exposure on BM tissue, expression of 4 genes encoding chemoattractive chemokines (CXCR4, CXCL9, CXCL10, CXCL12) were analyzed. BM from animals exposed to CS showed a significant reduction of CXCL9 expression levels when compared to sham-exposed animals (*p* < 0.05) (Fig. 7). Levels of CXCL10, CXCL12 and CXCR4 were unchanged (Fig. 7).

## Discussion

The results of the present study show in an experimental animal model, that short-term exposure to CS induces BM-MSCs dysfunction. Such dysfunction was observed in vivo*,* affecting the cell homing and proliferation capabilities of BM-MSCs in lungs exposed to CS and in vitro altering the rate of proliferation, senescence, differentiation and migration capacity.

One of the most relevant properties of BM-MSCs is their potential to be mobilized in response to tissue injury [2]. In this study, we evaluated how lung damage induced by CS exposure affects the recruitment capabilities of exogenously administrated BM-MSCs. Our results show that in this model, one month of CS exposure was sufficient to induce lung cellular damage and subsequent BM-MSCs mobilization. BM-MSCs administration was performed by two different routes, intratracheal instillation (IT) and by intravascular administration. Intravascular administration of BM-MSCs is commonly used in preclinical studies, due to ease of administration and broad dissemination [20]. However, IT instillation of BM-MSCs has been recently shown to attenuate lung damage [21, 22] and thus, no definite conclusion has been reached regarding the optimal administration route of BM-MSCs [23]. In our study, no significant differences were found between the two BM-MSCs administration routes used. Our results showed that regardless the administration route, higher numbers of BM-MSCs were recruited into CS-exposed animals compared to lungs of sham-exposed animals. Additionally, BM-MSCs homed into specific areas in the lung. They were primary found in the alveolar space and infiltrated into the alveolar septa. The airway epithelium of the lung is the major interface with the external environment. These results indicate that alveolar septa are an especially susceptible lung area readily exposed to CS. In line with our results, Rangasamy et al, showed a significant increase of cellular apoptosis at the alveolar septa, in CS-exposed mice lungs compared to sham-exposed mice lungs [24].

Exceptionally, very few BM-MSCs were detected within the adventitia of blood vessels even after intravascular administration of cells. This appears to suggest that longer CS exposure might be required to cause further vascular structural damage and promote BM-MSC mobilization into the vasculature. Liver and heart sections were also examined in order to identify the presence of labeled BM-MSCs. The number of cells counted in these tissues was low, only detected when cells were administrated intravascularly and consistently lower than the number of cells present in the lungs.

Although BM-MSCs isolated from CS-exposed animals presented homing capabilities as seen in BM-MSCs derived from sham-exposed animals, CS-exposed BM-MSCs showed a marked reduced capacity to engraft into the recipient’s lung when compared to BM-MSCs isolated from sham-exposed animals. Importantly, these results indicate that CS exposure compromised BM-MSC recruitment capacity. BM-MSCs obtained from CS-exposed animals showed lower proliferation and migration rate than BM-MSCs isolated from sham-exposed animals. Accordingly, Zhou et al, in a mice model of cigarette exposure presented in vivo and in vitro evidence that the number of recruited BM-MSCs in female uterus was significantly reduced in mice exposed to CS compared to sham-exposed mice [25].

In vitro, our results showed that BM-MSCs from non-exposed animals subjected to increasing concentrations of CSE had a significant reduction of both proliferative and migratory potential in a dose dependent manner. Accumulation of senescent BM-MSCs due to an increase concentration of CSE was detected by greater SA-β-gal activity. Osteogenic differentiation of BM-MSCs was also affected by CSE administration. In agreement with these results, previous studies have shown a reduced osteoblastic activity of BM-MSCs and an enhanced apoptosis when they were incubated with nicotine [26–30]. It has been shown that BM-MSCs express the alpha7 nicotinic receptor subunit and respond to nicotine in a dose-dependent manner [31, 32]. The impaired BM-MSCs proliferation, migration and recruitment capabilities observed in our CS animal model might be at least partly explained by these increased cellular aging and reduced BM-MSCs differentiation potential. It is commonly known that many factors in vivo can influence the assay result. In our series, cellular migration measured by the wound healing assay differs when assessed in vitro or vivo. Although both assays showed a distinct decrease of migration capacity in BM-MSCs from CS-exposed animals compared to sham-exposed BM-MSCs, it did not reach statistical significance in vivo. We hypothesize that the lack of significance in vivo assay is likely due to the interaction of many other factors such as tissue microenvironment and mechanical forces, secretion of cytokines, hormones and growth factors which might have increased the variability of the assay and influenced the end result. Moreover, patients with COPD present other co-morbidities such as alveoli damage which could restrict cellular regeneration capabilities.

The exact mechanisms by which BM-MSCs are selectively recruited in response to tissue damage are not known. To investigate the influence of CS in such recruitment mechanisms, we analyzed the expression of different chemokines and chemokine receptors in the bone marrow of CS-exposed guinea pigs. Key molecules involved in directing migration of cells are CXCR4 and its accompanying ligand CXCL12 [33, 34]. It is known that CXCL12 is a major chemotactic factor that promotes hematopoietic cell homing [35, 36]. It has been shown to enhance the migration of HSCs into ischemic myocardium and overexpression of CXCR4 in cultured BM-MSCs resulted in higher recruitment to acutely infarcted myocardium in rats [37]. However, controversial studies have also been reported. In an acute kidney injury mouse model, overexpression of CXCR4 in BM-MSCs did not result in an increase of BM-MSCs in the injured kidney and blockage of CXCR4 did not affect the intramyocardial migration of murine BM-MSCs to ischemic areas in mice [37]. Additionally, there was no correlation between serum CXCR4 levels and the number of circulating BM-MSCs [38]. In our experiments we could not find any changes regarding the expression of CXCR4 and CXCL12 in the BM of CS-exposed animals compared to sham-exposed animals. Therefore, we hypothesize that whilst CXCL12/CXCR4 signaling axis is involved in HSCs homing might not be mandatory for BM-MSCs migration and its role requires further investigation. Expression levels of other chemokines or growth factors including CXCL10, TGF-β or VEGF were not changed.

The mechanism of homing is a multistep process that includes migration of cells into the blood stream, transendothelial migration through the vascular walls and invasion into the target tissue [39]. The mechanism by which BM-MSCs is directed to the tissue and migrate across the endothelium, is not yet fully understood but it is likely that damaged tissue expresses specific receptors or ligands to facilitate traffic, adhesion and infiltration of BM-MSCs to the site of injury. C-X-C motif chemokine ligand 9 (CXCL9) is expressed by circulating BM-MSCs and it is known to play an important role in their migration through endothelium [40, 41].

In our study, CXCL9 expression levels were reduced in BM of CS-exposed animals compared to sham-exposed animals**.** Chamberlain et al, showed that higher levels of CXCL9 significantly enhanced BM-MSCs adhesion, crawling and transendothelial migration across murine aortic endothelial cells [41]. Thus, lower engraftment of CS exposed BM-MSCs into the GP lungs compared to BM-MSCs isolated from sham-exposed animals might be explained by lower CXCL9 expression. In our model, CXCL9 rather than CXCL12/CXCR4 signaling pathway seems to be involved in the homing of BM-MSCs.

However, due to availability reasons, the material used to measure CXCL9 expression was bone marrow from guinea pigs exposed to CS for 6 months rather than 1 month as previous experiments. Further short and long-term CS exposure studies are required to understand the insights of BM-MSCs mobilization in response to tissue injury and in particular the role of CXCL9 signaling pathway. The possibility to modulate the release of some key chemokines and subsequent BM-MSCs mobilization may be crucial in enhancing endogenous tissue regeneration.

### Limitations

In this study, it can not be confirmed that reduced proliferation rate seen in vitro at 4–12 days of culture, underpins the lower recruitment potential observed after 24 h of BM-MSCs administration in vivo. Thus, whilst reduced migration clearly plays a role, long term cellular viability and proliferation experiments are required to conclude. Additionally, changes on the expression of endothelial adhesion markers following CS exposure might be at least partly accountable for the impaired BM-MSCs recruitment potential. Unfortunately, those markers could not be measured due to the lack availability of guinea pig-specific antibodies for adhesion markers.

## Conclusion

In conclusion, defective lung repair in some diseases such as COPD might result, from an inadequate bone marrow contribution due to a lower mobilization or functional impairment of BM-derived circulating stem cells. This might provide a biological basis for the pathogenesis in COPD, in which BM-MSCs are dysfunctional and cannot provide adequate lung repair.

## Additional file

Additional file 1: Figure S1. Caspase 3 expression in BM-MSCs after stimulation with vehicle or different concentrations of CSE: BM-MSCs were incubated 24h with increasing concentrations of CSE. The graph shows the mean and the standard deviation of the relative gene expression (left graph). MTT-kit cell viability assay (Vybrant MTT cell proliferation assay kit) in. BM-MSCs after stimulation with vehicle or after different concentrations of CSE (right graph). All experiments were done in technical and biological triplicates. A *P* value of higher than 0.05 was considered not statistically significant (*P* > 0.05). (DOCX 131 kb)

## References

[1] Agustí A (2007). Systemic Effects of Chronic Obstructive Pulmonary Disease. Proc Am Thorac Soc.

[2] Leeman KT, Fillmore CM, Kim CF (2014). Lung stem and progenitor cells in tissue homeostasis and disease. Curr Top Dev Biol.

[3] Huertas A, Palange P (2011). Circulating endothelial progenitor cells and chronic pulmonary diseases. Eur Respir J.

[4] Pizarro S, Garcia-Lucio J, Peinado VI, Tura-Ceide O, Díez M, Blanco I, et al. Circulating progenitor cells and vascular dysfunction in chronic obstructive pulmonary disease. PLoS One. 2014; doi:10.1371/journal.pone.0106163. 10.1371/journal.pone.0106163 PMC4149524 25171153

[5] Fadini GP, Schiavon M, Cantini M, Baesso I, Facco M, Miorin M (2006). Circulating progenitor cells are reduced in patients with severe lung disease. Stem Cells.

[6] Palange P, Testa U, Huertas A, Calabrò L, Antonucci R, Petrucci E (2006). Circulating haemopoietic and endothelial progenitor cells are decreased in COPD. Eur Respir J.

[7] Kondo T, Hayashi M, Takeshita K, Numaguchi Y, Kobayashi K, Iino S (2004). Smoking cessation rapidly increases circulating progenitor cells in peripheral blood in chronic smokers. Arterioscler Thromb Vasc Biol.

[8] Michaud SE, Dussault S, Haddad P, Groleau J, Rivard A (2006). Circulating endothelial progenitor cells from healthy smokers exhibit impaired functional activities. Atherosclerosis.

[9] Boulais PE, Frenette PS (2015). Making sense of hematopoietic stem cell niches. Blood.

[10] Sage EK, Loebinger MR, Polak J, Janes SM (2008). The role of bone marrow-derived stem cells in lung regeneration and repair^*^ StemBook.

[11] Meirelles LS, Fontes AM, Covas DT, Caplan AI (2009). Mechanisms involved in the therapeutic properties of mesenchymal stem cells. Cytokine Growth Factor Rev.

[12] Bonnet D (2003). Biology of human bone marrow stem cells. Clin Exp Med.

[13] Schilders KA, Eenjes E, van Riet S, Poot AA, Stamatialis D, Truckenmüller R, et al. Regeneration of the lung: Lung stem cells and the development of lung mimicking devices. Respiratory Research.2016; doi: 10.1186/s12931-016-0358-z. 10.1186/s12931-016-0358-z PMC4842297 27107715

[14] Weiss DJ, Bertoncello I, Borok Z, Kim C, Panoskaltsis-Mortar A, Reynolds S (2011). Stem Cells and Cell Therapies in Lung Biology and Lung Diseases. Proc Am Thorac Soc.

[15] Sethi JM, Rochester CL (2000). Smoking and chronic obstructive pulmonary disease. Clin Chest Med.

[16] Brittan M, Hoogenboom MM, Padfield GJ, Tura O, Fujisawa T, Maclay JD, et al. Endothelial Progenitor Cells in Patients with Chronic Obstructive Pulmonary Disease. Am.J.Physiol Lung Cell Mol.Physiol. 2013; doi: 10.1152/ajplung.00183.2013. 10.1152/ajplung.00183.2013 PMC3882533 24142520

[17] Nadri S, Soleimani M, Hosseni RH, Massumi M, Atashi A, Izadpanah R (2007). An efficient method for isolation of murine bone marrow mesenchymal stem cells. Int J Dev Biol.

[18] Pal R, Hanwate M, Jan M, Totey S (2009). Phenotypic and functional comparison of optimum culture conditions for upscaling of bone marrow-derived mesenchymal stem cells. J Tissue Eng Regen Med.

[19] Livak KJ, Schmittgen TD (2001). Analysis of relative gene expression data using real-time quantitative PCR and the 2(-Delta Delta C(T)) Method. Methods.

[20] Cruz FF, Borg ZD, Goodwin M, Sokocevic D, Wagner DE, Coffey A (2015). Systemic Administration of Human Bone Marrow-Derived Mesenchymal Stromal Cell Extracellular Vesicles Ameliorates *Aspergillus* Hyphal Extract-Induced Allergic Airway Inflammation in Immunocompetent Mice. Stem Cells Transl Med.

[21] Guan XJ, Song L, Han FF, Cui ZL, Chen X, Guo XJ (2013). Mesenchymal stem cells protect cigarette smoke-damaged lung and pulmonary function partly via VEGF-VEGF receptors. J Cell Biochem.

[22] Ingenito EP, Tsai L, Murthy S, Tyagi S, Mazan M, Hoffman A (2012). Autologous lung-derived mesenchymal stem cell transplantation in experimental emphysema. Cell Transplant.

[23] Antunes MA, Abreu SC, Cruz FF, Teixeira AC, Lopes-Pacheco M, Bandeira E (2014). Effects of different mesenchymal stromal cell sources and delivery routes in experimental emphysema. Respir Res.

[24] Rangasamy T, Misra V, Zhen L, Tankersley CG, Tuder RM, Biswal S.Cigarette smoke-induced emphysema in A/J mice is associated with pulmonary oxidative stress, apoptosis of lung cells, and global alterations in gene expression. Am J Physiol Lung Cell Mol Physiol. 2009; doi: 10.1152/ajplung.90369.2008. Epub 2009 Mar 13. 10.1152/ajplung.90369.2008 PMC2692799 19286929

[25] Zhou Y, Gan Y, Taylor HS. Cigarette smoke inhibits recruitment of bone-marrow-derived stem cells to the uterus. Reprod Toxicol. 2011;doi: 10.1016/j.reprotox.2010.10.007. 10.1016/j.reprotox.2010.10.007 PMC3207965 20955787

[26] Kim DH, Liu J, Bhat S, Benedict G, Lecka-Czernik B, Peterson SJ (2013). Peroxisome proliferator-activated receptor delta agonist attenuates nicotine suppression effect on human mesenchymal stem cell-derived osteogenesis and involves increased expression of heme oxygenase-1. J Bone Miner Metab.

[27] Hoogduijn MJ, Cheng A, Genever PG (2009). Functional nicotinic and muscarinic receptors on mesenchymal stem cells. Stem Cells Dev.

[28] Ng TK, Carballosa CM, Pelaez D, Wong HK, Choy KW, Pang CP (2013). Nicotine alters MicroRNA expression and hinders human adult stem cell regenerative potential. Stem Cells Dev.

[29] Wang XJ, Liu YF, Wang QY, Tsuruoka M, Ohta K, Wu SX (2010). Functional expression of alpha 7 nicotinic acetylcholine receptors in human periodontal ligament fibroblasts and rat periodontal tissues. Cell Tissue Res.

[30] Gullihorn L, Karpman R, Lippiello L (2005). Differential effects of nicotine and smoke condensate on bone cell metabolic activity. J Orthop Trauma.

[31] Deng Y, Li TQ, Yan YE, Magdalou J, Wang H, Chen LB (2012). Effect of nicotine on chondrogenic differentiation of rat bone marrow mesenchymal stem cells in alginate bead culture. Biomed Mater Eng.

[32] Schraufstatter I, DiScipio RG, Khaldoyanidi S (2010). Alpha 7 subunit of nAChR regulates migration of human mesenchymal stem cells. J Stem Cells.

[33] Phillips RJ, Burdick MD, Hong K, Lutz MA, Murray LA, Xue YY (2004). Circulating fibrocytes traffic to the lungs in response to CXCL12 and mediate fibrosis. J Clin Invest.

[34] Xu J, Mora A, Shim H, Stecenko A, Brigham KL, Rojas M (2007). Role of the SDF-1/CXCR4 axis in the pathogenesis of lung injury and fibrosis. Am J Respir Cell Mol Biol.

[35] Broxmeyer HE, Orschell CM, Clapp DW, Hangoc G, Cooper S, Plett PA (2005). Rapid mobilization of murine and human hematopoietic stem and progenitor cells with AMD3100, a CXCR4 antagonist. J Exp Med.

[36] Mendez-Ferrer S, Lucas D, Battista M, Frenette PS (2008). Haematopoietic stem cell release is regulated by circadian oscillations. Nature.

[37] Zhao RC. (ed.). Essentials of Mesenchynal Stem Cell Biology and its Clinical Translation. ©Springer Science+Business Media Dordrecht 2013. doi:10.1007/978-94-007-6716-4_1.

[38] Wu Y, Zhao RC (2012). The Role of Chemokines in Mesenchymal Stem Cell Homing to Myocardium. Stem Cell Rev.

[39] Kholodenko IV, Konieva AA, Kholodenko RV, Yarygin KN (2013). Molecular mechanisms of migration and homing of intravenously transplanted mesenchymal stem cells. J Regen Med Tissue Eng.

[40] Ahmed SK, Soliman AA, Omar SM, Mohammed WR (2015). Bone Marrow Mesenchymal Stem Cell Transplantation in a Rabbit Corneal Alkali Burn Model (A Histological and Immune Histo-chemical Study). Int J Stem Cells.

[41] Chamberlain G, Smith H, Rainger GE, Middleton J, et al. Mesenchymal stem cells exhibit firm adhesion, crawling, spreading and transmigration across aortic endothelial cells: effects of chemokines and shear. PLoS One. 2011;doi: 10.1371/journal.pone.0025663. 10.1371/journal.pone.0025663 PMC3182247 21980522

