# Supplementary material for: Cigarette smoke challenges bone marrow mesenchymal stem cell capacities in guinea pig
Source: Respir Res. 2017 Mar 23;18:50. doi: 10.1186/s12931-017-0530-0 (PMC5363047; doi:10.1186/s12931-017-0530-0)
Supplement: Additional file 1: Figure S1. — Caspase 3 expression in BM-MSCs after stimulation with vehicle or different concentrations of CSE: BM-MSCs were incubated 24h with increasing concentrations of CSE. The graph shows the mean and the standard deviation of the relative gene expression (left graph). MTT-kit cell viability assay (Vybrant MTT cell proliferation assay kit) in. BM-MSCs after stimulation with vehicle or after different concentrations of CSE (right graph). All experiments were done in technical and biological triplicates. A P value of higher than 0.05 was considered not statistically significant (P > 0.05). (DOCX 131 kb) [file 12931_2017_530_MOESM1_ESM.docx]

***Response to reviewers***

The authors are grateful for the positive response to their manuscript and are pleased to address the specific points raised by the reviewers.

***Reviewer 1:***

*<This is an extremely thorough and valuable study that adds significantly to the field. The studies are well conducted and the conclusions are sound. I have only a few mostly minor concerns>*

***Comments:***

***Question 1:***

**1. A point of discussion is that the *in vitro* findings do not necessarily relate to the *in vivo* findings. Reduced proliferation is seen *in vitro* after 4-12 days but the *in vivo* studies used only a 24h time window. It is not likely therefore that proliferation underpins the *in vivo* effects, though migration may clearly play a role. This should be discussed.**

We agree with the reviewer’s comment and migration and proliferation processes have to be distinguished accordingly. The main objective of the manuscript is to assess the recruitment (migration) capabilities of exogenously administrated BM-MSCs and our results indicate that CS exposure compromised BM-MSC recruitment capacity. As the reviewer correctly points out, this was assessed on the 24h time window after the exogenously administration of BM-MSCs. The design of our study was a short-term monitoring of immediate cell engraftment to avoid possible false-positive fluorescent signals. *In vivo* tracking of transplanted cells over time is complex. Rapid exocytosis of administrated cells by live cells such as macrophages has been observed as early as 48h post-transplantation and may generate false-positive results.

Certainly, proliferation was not specifically studied *in vivo* and the reduced proliferation rate seen *in vitro* (4-12 days) might not necessarily correlate with the *in vivo* results.

We have added an explanation in the manuscript’s limitation section to further clarify this issue.

*In this study, it can not be confirmed that reduced proliferation rate seen in vitro at 4-12 days of culture, underpins the lower recruitment potential observed after 24h of BM-MSCs administration in vivo. Thus, whilst reduced migration clearly plays a role, long term cellular viability and proliferation experiments are required to conclude.* **Page 15, Lines 357-360.**

***Question 2:***

**2. A related point is the expression of adhesion markers. Presumably the homing of BMMSCS to the lung (and more so in the CS exposed lung) has to do with changes in local endothelial cell activation. The study would benefit from analysis of adhesion markers in the various models or at the very least from discussion on this aspect.**

The authors’ thank you for the comment and the following sentence has been added in the limitation section.

*Additionally, changes on the expression of endothelial adhesion markers following CS exposure might be at least partly accountable for the impaired BM-MSCs recruitment potential. Unfortunately, those markers could not be measured due to the lack availability of guinea pig-specific antibodies for adhesion markers.* **Page 15, Lines 360-364.**

The authors have experience analyzing other species antibodies for adhesion markers such as mouse antibodies, but unfortunately there is not cross-reactivity and can not be used.

***Question 3:***

**3. The protocol used for the CS exposure of BMMSCS donor guinea pigs is not clear to me. Was this 4 week as for the CS exposed animals or 6 months as mentioned at one point in the paper?**

Animals from the CS groups were exposed to cigarette smoke 5 days/week during 4 weeks. For *in vivo* gene expression analysis, lungs from sham and 4 weeks CS-exposure were used. As a result of the previous results, in a second set of experiments aiming to understand the role of CXCL9, bone marrow of guinea pigs exposed to CS for 6 months were used.

To clarify this, we have added the following explanation in our discussion.

*However, due to availability reasons, the material used to measure CXCL9 expression was bone marrow from guinea pigs exposed to CS for 6 months rather than 1 month as previous experiments. Further short and long-term CS exposure studies are required to understand the insights of BM-MSCs mobilization in response to tissue injury and in particular the role of CXCL9 signaling pathway.* **Page 15, Lines 349-353.**

***Question 4:***

**4. What marker was measured in the senescence assay? Beta galactosidase?**

It is now detailed in the methods section that the Senescence Cell Histochemical Staining Kit from Sigma-Aldrich measures the number of senescent cells using an assay based on a histochemical stain for β-galactosidase activity. **Page 7, Lines 162-163.**

***Question 5:***

**5. Please add legend labels to figure 5.**

Legend labels in figure 5 have now been added.

***Reviewer 2***

*<This paper shows that (short-term) cigarette smoke exposure induces BM-MSC dysfunction in a guinea pig model. This dysfunction was found with in vitro exposure to cigarette smoke as well as with in vivo exposure. The dysfunction found, predominantly affected the cell homing and proliferation in the in vivo exposed BM-MSCs. Whereas in the in vitro cigarette exposure, this dysfunction also entailed dysfunction in senescence, differentiation and migration of the BM-MSCs. Moreover, in cigarette smoke exposed guinea pigs the migration of BM-MSCs towards the lungs, from the systemic circulation (vascular administration) is impaired in the BM-MSCs, which were exposed to cigarette smoke compared to healthy BM-MSCs. This could possibly provide a biological basis for the pathogenesis in COPD, in which BM-MSCs are dysfunctional and cannot provide adequate lung repair.
I believe the hypothesis is interesting, promising and of interest for publication in Respiratory Research. The authors have performed an extensive analysis of the functional capabilities of the BM-MSCs and have used an comprehensive method to study said BM-MSCs, I do, however, have some issues, which I will specify below.>*

***Comments (major):***

***Question 1:***

**In the introduction (Background) page 2, line 49 - 52, the authors mention that progenitor cells present in the bone marrow are essential in tissue maintenance and restoration of the normal function as a result of tissue damage. However, how much is known about the regeneration of lung tissue in general (in patients/animal models) without the influence of cigarette smoke? For instance, is regeneration of lung tissue possible in emphysematous diseases such as poorly regulated asthma, alpha 1 anti-trypsin deficiency, or damage due to ventilation, with functional BM-MSCs. This could be mentioned in the Discussion.**

Lung tissue has a low level of cell regeneration during homeostasis but is rather good at regenerating itself after injury. Lung regeneration involves activation of progenitor cells as well as the proliferation of healthy adjacent cells and different subsets of progenitor cells residing in the lung’s specialized niches. Disruption of this regeneration potential is the cause of several lung diseases such as COPD.

As recently reviewed by Weiss *et al*., 2011 and Schilders *et al*., 2016, studies of adult stem and progenitor cells, notably MSCs derived from bone marrow and other tissues, have increasingly provided evidence of safety and efficacy in animal models of acute and fibrotic lung injuries as well as in asthma, bronchopulmonary dysplasia, COPD, sepsis, and other lung diseases and human lung explant models of COPD. The mechanisms underlying the effects of MSCs are not fully understood but growing evidence suggests that paracrine and immunomodulatory effects released by the MSCs are likely the responsible of the observed beneficial effects of MSC therapy.

Following the reviewer’s suggestion, we have added the following text in the introduction section of the manuscript.

*Studies of BM-MSCs have provided evidence of safety and efficacy in animal and human lung explant models of acute and fibrotic lung injuries as well as in asthma, bronchopulmonary dysplasia, COPD, sepsis, and other lung diseases [13, 14]. There is no clear evidence of the potential role of these cells on the regeneration of lung tissue in these diseases. However, growing evidence suggests that paracrine and immunomodulatory effects released by the BM-MSCs are likely the responsible factors of the beneficial effects of MSC therapy [13, 14].* **Page 2-3, Lines 64-70.**

These references were included accordingly.

*[13] Schilders KA, Eenjes E, van Riet S, Poot AA, Stamatialis D, Truckenmüller R, et al. Regeneration of the lung: Lung stem cells and the development of lung mimicking devices. Respiratory Research.2016; doi: 10.1186/s12931-016-0358-z.*

*[14] Weiss DJ, Bertoncello I, Borok Z, Kim C, Panoskaltsis-Mortar A, Reynolds S, et al. Stem Cells and Cell Therapies in Lung Biology and Lung Diseases.* [*Proc Am Thorac Soc*](https://www.ncbi.nlm.nih.gov/pmc/articles/PMC3132784/)*. 2011; 8:223.*

***Question 2:***

**2. The scratch assay, which was used, is an interesting way to determine the regenerative function of the BM-MSCs. However, I wonder how this applies to damage in alveoli. When the walls of alveoli rupture the sac would collapse in on itself, making regeneration more difficult, I can imagine. The authors should mention this as a limitation of the scratch assay (Limitation section see below). This does not take away I find the analysis interesting and definitely worth mentioning.**

The scratch assay is an easy and well-developed method to essentially measure cell migration *in vitro.* In our model, we aimed to study whether CS exposure had an *in vitro* effect on BM-MSCs migration. Indeed, patients with damaged alveoli or other systemic co-morbidities may present a further reduction in their regenerative potential.

Following the reviewer’s suggestion, we have added an explanation in the discussion section of the manuscript.

*It is commonly known that many factors in vivo can influence the assay result. In our series, cellular migration measured by the wound healing assay differs when assessed in vitro or vivo. Although both assays showed a distinct decrease of migration capacity in BM-MSCs from CS-exposed animals compared to sham-exposed BM-MSCs, it did not reach statistical significance in vivo. We hypothesize that the lack of significance in vivo assay is likely due to the interaction of many other factors such as tissue microenvironment and mechanical forces, secretion of cytokines, hormones and growth factors which might have increased the variability of the assay and influenced the end result. Moreover, patients with COPD present other co-morbidities such as alveoli damage which could restrict cellular regeneration capabilities.* **Page 13, Lines 302-312.**

***Question 3:***

**3. There needs to be a limitation section in the Discussion.**
Following the indications of the Reviewer, we have added a limitation section in the discussion. **Page 15, Lines 356-364.**

***Question 4:***

**4. The number of samples for the analyses in the individual groups of the CS exposed guinea pigs are relatively small (n=6-8)**

We agree with the reviewer’s comment that a greater number of animals would have strengthened our main results. However, these number of animals used was a limitation imposed by the complexity of these studies. Additionally, the following reasoning was applied to calculate the sample size of the study. The main endpoint of this study was the difference in the number of infiltrated cells in the lung of sham exposed as compared with CS exposed animals. Taken data from a previous pilot study with 4 animals, we determined the range of values of infiltrated cells in the total lung. Accordingly, assuming a standard deviation of 1 in the number of cells, a sample size of 8 subjects per group was needed to detect a minimal difference of 2 between groups; with 95% power and a two-tailed p-value less than 0.05. The calculated sample size fitted well with the maximal capacity of our facilities.

***Question 5:***

**5. In Page 8, Line 201-202 it states that BM-MSC preferentially locate at the alveolar septum, (this is mentioned various times, as well as in the discussion) and that this is based on Fig 3b. However, from Fig 3b I cannot distil that the cells predominantly locate in the alveolar septum. Fig 3b shows that there is a difference in infiltration of BM-MSCs based on smoking status of the guinea pigs. I do not see how you determined they predominantly locate in the alveolar septum based on fig 3b, please explain. Moreover, I do not understand how you calculated the p-value.**

The authors apologize for this misunderstanding. When we wrote that BM-MSC preferentially located at the alveolar septum, we meant that most cells found in the lung tissue located at alveolar septum (10 times more) when compared to the cells found in structures like pulmonary arteries.

To clarify this question, we have added the following sentence:

*BM-MSC preferentially locate at the alveolar septum when compared to the number of cells found in the pulmonary arteries.* **Page 9, Lines 205-206.**

The method which calculates the p value is explained in question 6.

***Question 6:***

**6. Mention method of calculating the p-value in figure legends 3b, 4, 5, 6, 7.**

Following the Reviewer indications, we have reviewed the statistical method analysis and included the statistic method applied in each figure legend.

*Figure 3B: b) Box plots of infiltrated BM-MSC in total lung and in the alveolar septa. P value was assessed by Mann-Whitney U-test.* **Page 25, Lines 560**.

*Figure 4: Administration of BM-MSC obtained from healthy or CS exposed animals. P value was assessed by Mann-Whitney U-test.* **Page 25, Lines 564-565.**

*Figure 5. Cell growth kinetics of BM-MSCs. P value was assessed by Two way ANOVA Repeated Measures. *denotes P<0.05.* **Page 25, Lines 573-574.**

*Figure 6.* *In vitro* effects of CSE in cultured BM-MSC. *P value was assessed by one-way ANOVA and post-hoc Holm-Sidak method.* **Page 26, Lines 587-588.**

*Figure 7: Expression of 6 animals. Reactions were run in triplicates Relative gene expression was analyzed by 2-ΔΔct-method. Significant differences were assessed using the Mann-Whitney U-test * p<0.05 vs control.* **Page 26, Lines 594-595.**

***Question 7:***

**7. In Page 9 line 204 - 205 you state that CS-exposed animals showed greater CS-derived BM-MSC lung infiltration compared to lungs of sham-exposed animals, however no significance is shown about this in figure 4. Based on fig 4 I can imagine it being statistically significant, however I would like to see a calculation of significance.**

We apologize for this misunderstanding. This information is shown in Figure 3 instead of Figure 4. **Page 9, Line 209.**

***Question 8:***

**8. I would like tables in the results section on the ANOVA calculations and the post hoc analyses, and not only bar-graph figures.**

We consider that presenting raw data as median and range interquartile is an easier approach for data interpretation. Accordingly, we performed a Kruskal Wallis analysis and a Mann Whitney U test for the comparison within groups for each factor (Factor 1: CS exposed animals, Factor 2: CS-exposed BM-MSCs). Following the reviewer’s suggestion we added the results of the Kruskal Wallis analysis in a table form (Table 1). Mann Whitney U test results are shown in figures 3B and 4.

Additionally, we have also included a Two-Way ANOVA analysis after a logarithmic data transformation to adjust normalization (Factor 1: CS exposed animals, Factor 2: CS-exposed BM-MSCs). For post hoc analysis we apply the Holm-Sidak test. The results of Two Way ANOVA are not shown in the text to avoid data duplication. However, if the reviewer and/or editors consider appropriate to include it, we would be happy to add it.

The following modifications have been made in the text:

*Results are expressed as mean ± SD or median and interquartile range, depending on the parametric or non-parametric distribution. Comparisons between groups were performed using an analysis of variance (ANOVA) or the Kruskal-Wallis test, when appropriate. Post hoc comparisons were done using Mann-Whitney U-test. Significant differences were considered at p<0.05 level. All analyses were performed using SigmaPlot v.11.0 software (Systat Software Inc., Chicago, IL).* **Page 8, Lines 184-189.**

**Table 1**

|  | **Experimental groups** | | | | **P value Kruskall-Wallis** |
| --- | --- | --- | --- | --- | --- |
|  | **ShA/HMSC** | **ShA/CSMSC** | **CSA/HMSC** | **CSA/CSMSC** |  |
|  | **Intratracheal Administration** | | | |  |
| **MSC in total lung** | 1.450  1.294 – 1.996 | 1.688  1.525 – 1.725 | 3.700  3.25 – 3.975 | 3.05  2.569 – 3.256 | <0.001 |
| **MSC in Alveolar septa** | 0.300  0.175 – 0.569 | 0.388  0.375 – 0.500 | 0.988  0.600 – 1.375 | 0.650  0.350 – 0.825 | 0.031 |
| **MSC in pulmonary arteries** | 0.000  0.000 – 0.038 | 0.037  0.000 – 0.075 | 0.000  0.000 – 0.044 | 0.00  0.000 – 0.000 | 0.641 |
|  | **Intravascular Administration** | | | |  |
| **MSC in total lung** | 1.813  1.450 – 2.450 | 1.337  1.125 – 1.663 | 3.264  2.792 – 4.105 | 2.612  2.362 – 3.003 | <0.001 |
| **MSC in Alveolar Septa** | 1.038  0.725 – 1.200 | 0.200  1.125 – 0.225 | 1.175  1.063 – 1.475 | 0.463  0.362 – 0.700 | <0.001 |
| **MSC in pulmonary arteries** | 0.025  0.000 – 0.050 | 0.075  0.063 – 0.088 | 0.125  0.031 – 0.181 | 0.087  0.025 – 0.138 | 0.362 |

**Table 1. Number of cells by microscopic field. Data are presented as median and range interquartile. A p-value <0.05 was considered significant. ShA: Sham exposed animals; HMSC: MSC from healthy animals; CSA: CS exposed animals; CSMSC: MSC from CS exposed animals.**

Two-Way ANOVA analysis after a logarithmic data transformation to adjust normalization (Factor 1: CS exposed animals, Factor 2: CS-exposed BM-MSCs). For post hoc analysis we apply the Holm-Sidak test

|  | **Experimental groups** | | | |  | **Two-way ANOVA main effects p-value** | | |
| --- | --- | --- | --- | --- | --- | --- | --- | --- |
|  | **ShA/HMSC** | **ShA/CSMSC** | **CSA/HMSC** | **CSA/CSMSC** |  | **Animal CS exposure** | **BM MSC CS exposure** | **Interaction** |
|  |  |  | **Intratracheal administration** | | |  |  |  |
| **MSC in total lung** | 0.419±0.102 | 0.543±0.110 | 1.345±1.020 | 1.044±0.102 |  | <0.001 | 0.408 | 0.054 |
| **MSC in Alveolar septa** | -1.303±0.232 | -0.813±0.251 | -0.131±0.251 | -0.597±0.232 |  | 0.009 | 0.959 | 0.060 |
| **MSC in pulmonary arteries** | ND | ND | ND | ND |  |  |  |  |
|  |  |  | **Intravascular Administration** | | |  |  |  |
| **MSC in total lung** | 0.610±0.110 | 0.347±0.095 | 1.218±0.102 | 0.954±0.090 |  | <0.001 | 0.015 | 0.995 |
| **MSC in Alveolar Septa** | -0.191±0.197 | -1.664±0.170† | 0.220±0.182 | -0.697±0.170 |  | <0.001 | <0.001 | 0.135 |
| **MSC in pulmonary arteries** | ND | ND | ND | ND |  |  |  |  |

Number of cells by microscopic field. Logarithmic scale. Data are presented as mean±SEM. A p-value <0.05 was considered significant. ShA: Sham exposed animals; HMSC: MSC from healthy animals; CSA: CS exposed animals; CSMSC: MSC from CS exposed animals; ND: Non determined

***Question 9:***

**9. Can you explain why the wound healing assay is significantly different in the in vitro but not in *in vivo* CS exposed BM-MSC cells? Please explain in the discussion.**

The authors share the same puzzling question with the reviewer. However, commonly *in vitro* experimental results are not always equivalent to the *in vivo* findings. *In vivo* many factors such as the cellular microenvironment directly affect conditions around the cellular types via de secretion of cytokines, hormones, or growth factors as well as mechanical forces caused by the movement of the organism or the movement of the physiological fluids such as blood. All these factors are especially difficult to control and significantly influence the result, the assay variability and the error. Although our results showed a distinct decrease of migration capacity in BM-MSCs from CS-exposed animals compared to sham-exposed BM-MSCs, it did not reach statistical significance *in vivo* (p=0.28). Accordingly, we have added the following explanation in the discussion section of the manuscript.

*It is commonly known that many factors in vivo can influence the assay result. In our series, cellular migration measured by the wound healing assay differs when assessed in vitro or vivo. Although both assays showed a distinct decrease of migration capacity in BM-MSCs from CS-exposed animals compared to sham-exposed BM-MSCs, it did not reach statistical significance in vivo. We hypothesize that the lack of significance in vivo assay is likely due to the interaction of many other factors such as tissue microenvironment and mechanical forces, secretion of cytokines, hormones and growth factors which might have increased the variability of the assay and influenced the end result.* **Page 13, Lines 302-310.**

***Question 10:***

**10. Interesting is that bone marrow tissue from CS-exposed animals showed a reduction of CXCL9 expression compared to sham-exposed animals. Is it possible to provide a hypothesis on why CXCL9 expression is reduced but expression of CXCR4, CXCL10 and CXCL 12 is not?**

Cellular homing is a complex multistep process and the mechanism which BM-MSCs are recruited in response to an injury is not fully understood. Aiming to clarify this issue we studied the expression of different chemokines and *chemokine* receptors such as CXCR4, CXCR12, CXCL10 and CXCR9. Our results showed no significant difference on the levels of CXCR4, CXCR12, and CXCL10 in the BM of CS-exposed animals compared to sham-exposed animals.

Studies investigating the cell homing capabilities of hematopoietic stem cells (HSCs) in the bone marrow have clearly shown that CXCL12/CXCR4 signaling axis plays an important role in the migration of HSCs in response to tissue damage (31, 32). It has also been demonstrated in several reports that CXCL12 is a major chemotactic factor that promotes hematopoietic cell homing (33, 34). However, there have been controversial results about the expression of CXCR4 and CXCR12 in BM-MSCs. This issue is stated and referenced in the discussion section of the manuscript page 13 line 316-321.

We hypothesize that whilst CXCL12/CXCR4 signaling axis is involved in HSCs homing might not be mandatory for BM-MSCs migration.

In contrast, CXCL9 expression levels were reduced in BM of CS-exposed animals compared to sham-exposed animals. CXCL9 seems to be involved in enhancing BM-MSCs adhesion, crawling and transendothelial migration (39). Thus, lower engraftment of CS exposed BM-MSCs into the animal lungs might be explained by lower CXCL9 expression.

We have now modified the discussion section specifying and expanding our hypothesis:

*We hypothesize that whilst CXCL12/CXCR4 signaling axis is involved in HSCs homing might not be mandatory for BM-MSCs migration and its role requires further investigation.* **Page 14, line 328-330.**

*In our model, CXCL9 rather than CXCL12/CXCR4 signaling pathway seems to be involved in the homing of BM-MSCs.* **Page 14, line 345-346.**

***Question 11:***

**11. The conclusion makes a too large of a statement based on the available data.
"CS has been related to many significant extrapulmonary events such as cardiovascular diseases or reproductive effects in addition to many forms of cancer. Considering the results of this study, the effects of CS on BM-MSCs might be considered a general mechanism by which smoking leads to long term organ damage through defective tissue repair mechanisms."
This claim is too grand, the regenerative capacities of different organs differ greatly, this article only studies lung samples (and vascular samples, however, no significance). Or mention in discussion that this is a possible general mechanism, but not in the conclusion.**

The author’s thank you for the comment and the sentence in the conclusion section has been revised and changed accordingly.

*In conclusion, defective lung repair in some diseases such as COPD might result, from an inadequate bone marrow contribution due to a lower mobilization or functional impairment of BM-derived circulating stem cells. This might provide a biological basis for the pathogenesis in COPD, in which BM-MSCs are dysfunctional and cannot provide adequate lung repair*. **Page 15 line 367-370.**

***Comments (minor):***

***Question 1:***

**1. I found which samples were used in the methods section (which was very comprehensive) somewhat difficult to follow.**

Following the reviewer comment, we have revised the methods section.

***Question 2:***

**2. Bar graphs from fig 3b are also visible in fig 4.**

We have modified figure 4 accordingly to statistical analysis applied.

***Question 3 and 4:***

**3. Interesting is that the degree of infiltration towards the lungs in CS guinea pigs is dependent on whether the BM-MSCs were exposed to CS (fig 4B) with vascular administration. However, with tracheal administration (fig 4A) this is not significantly different. Can you offer an explanation to why this is? Can you explain why there is a significant difference in fig 4 B (alveolar septum infiltration) in SHAM animals between CS-MSC and Healthy MSC?**

The author’s thank the reviewer observation. It is certain that higher degree of cellular infiltration to the alveolar septum was found via vascular administration than via tracheal administration. At present, and based on the results presented here, we can only hypothesize that in sham animals, intravascular administration might present better traffic towards the alveolar septum than via tracheal administration. This might be because intravascular route might provide broad cellular distribution and less variability between samples compared to intratracheal instillation. In order to be able to confirm these results, further studies are required.

***Question 5:***

**5. Line 263 the abbreviation IT has not been used before in the article and should be spelled out.**

The author’s thank you for the comment and the sentence has been revised. **Page 11, line 261.**

***Question 6:***

**6. On Page 278 - 281 new data is provided in the discussion which has not been mentioned in the results section. Please add this to result section before discussing it in the Discussion. Or remove all together.**

We agree and this has now been removed from the manuscript. **Page 12, line 278-281.**

***Question 7:***

**7. Page 14, line 341 the sentence is not correctly formulated ("therefore" should either be removed or placed elsewhere in the sentence):
"In conclusion, defective lung repair in some diseases such as COPD might result therefore, from an inadequate bone marrow contribution due to a lower mobilization etc."
Change to, for instance:
In conclusion, defective lung repair in some diseases such as COPD might result from an inadequate bone marrow contribution etc...**

The authors’ thank you for the comment and the sentence has been modified accordingly. **Page 15 line 367-370.**

***Question 8:***

**8. The vascular administration of BM-MSCs (healthy/CS-BM-MSCs) were administered via cardiac puncture. Page 8, line 199. I was just wondering whether this does not kill the guinea pig, as I have never performed this before (this question is just for my own understanding).**

Vascular administration via cardiac puncture, under general anesthesia, is a useful way to inject large volumes into circulation or for blood collection in guinea pigs and hamsters (See Laboratory Animals 27:1-22 (1993)) causing minimal damage to the myocardium. The most important advantage is that does not require surgery and therefore the recovering after anesthesia is quick, safe and free of potential infections. Although procedures may require some technical practice, usually good results are achieved after few attempts. Briefly, with the anesthetized animal in supine position, we use a needle of 20G which is inserted vertically (90 degrees within the horizontal axis) within the second and third rib (left side), always next to the sternum (very important to avoid the lung when introduce the needle). Then, the needle is inserted until you sense the heart (the heartbeats are very strong) and the embolus of syringe is suck up. If you are into the ventricle, the blood should flow into the syringe’s body. At this moment you can inject the physiological serum containing the cells of interest.
